# The Italian breakthrough in CRISPR trials for rare diseases: a focus on beta-thalassemia and sickle cell disease treatment

**DOI:** 10.3389/fmed.2024.1356578

**Published:** 2024-02-15

**Authors:** Francesca Greco, Marco Cosentino, Franca Marino

**Affiliations:** Center for Research in Medical Pharmacology, University of Insubria, Varese, Italy

**Keywords:** CRISPR, rare disease, clinical trial, beta thalassaemia, sickle cell disease

## Abstract

The development of gene therapy and the current advantageous method of clustered regularly interspaced short palindromic repeats (CRISPRs) has allowed the implementation of several clinical trials aimed at studying the possible efficacy of gene therapy for rare diseases. Rare diseases pose a global challenge, in that their collective impact on health systems is considerable, whereas their individually rare occurrence hinders research and development of efficient therapies. Despite the low prevalence of individual rare diseases, there are more than 7,000 defined rare diseases affecting 3.5–5.9% of the global population. Rare diseases are mostly chronic and approximately 80% are caused by genetic mutation with an early-life onset. In Italy, in 2021 were recorded more than 400,000 people with rare disease. Because of its location and history, Italy has an unfortunate statistic regarding the presence and prevalence of two rare genetic diseases, namely beta-thalassemia, of which there are about 90 million carriers worldwide, 400,000 of whom are actually affected, and sickle cell disease, with about 300 million carriers and 6.5 million people affected worldwide. Advancements in genomic studies allowed Italy to join clinical trials to study effective and resolving gene therapies for BT and SCD. This study reports on the impact of rare diseases in Italy, ongoing studies, and recent achievements in BT and SCD trials using the CRISPR method and remaining hurdles in the application of CRISPR technology to rare diseases, also taking a glimpse at the newest challenges and future opportunities in the genetic treatment for rare diseases.

## 1 Introduction

CRISPRs (clustered regularly interspaced short palindromic repeats) are short DNA sequences with unique spacer sequences that, along with CRISPR-associated (Cas) proteins, constitute an adaptive immune system in many bacteria and archaea against invading bacteriophages (1). By using short RNA molecules as a template, Cas highly makes sequences specific cuts in DNA molecules that can be exploited to insert genes, or to precisely modify the nucleotide sequence at the cut site.

The distinction between CRISPR and gene therapy is crucial in the field of genetic medicine. CRISPR is a specific genome-editing technology that enables precise modification of DNA sequences. In contrast, gene therapy encompasses a broader range of strategies aimed at treating genetic disorders by introducing, altering, or repairing genes within an individual’s cells. While CRISPR is a powerful tool within the scope of gene therapy, it is just one of many techniques employed to achieve therapeutic genetic modifications (2).

Compared with conventional gene-editing tools [zinc-finger nucleases (ZFNs) and transcription activator-like effector nucleases (TALENs)], CRISPR-Cas9 is easier and faster, because the system requires only one Cas9 endonuclease and a short single gRNA (3).

CRISPR technology holds immense potential in treating rare genetic disorders by precisely editing faulty genes. Its ability to correct underlying genetic mutations offers hope for targeted and personalized therapies, addressing the root cause of rare diseases rather than just managing symptoms. CRISPR opens new avenues for effective treatments and potential cures, revolutionizing the management of rare conditions worldwide.

As never before, it is now possible to survey currently ongoing clinical trials using a publicly available database to obtain useful information on current trends in rare diseases research, providing a unique vantage point for researchers, medical professionals, and even the general public to gain valuable insights.

The development, manufacturing, evaluation, and accessibility of these innovative genome editing products present novel challenges, as previously outlined in academic publications and discussed during a European Medicines Agency (EMA) expert meeting in 2017. The “Genome editing EU-IN Horizon Scanning Report” (EMA/319248/2020) (4) released in 2021 focuses on the regulatory perspective of *ex*- and *in vivo* genome editing as medicinal products for human patients and anticipates changes in this landscape over the next decade. The report contains the EMA’s Regulatory Science Strategy to 2025 and the European Medicines Agencies Network (EMRN) Strategy to 2025 that emphasize collaboration and a forward-looking approach to regulatory science. One primary objective is to enhance the scientific quality of evaluations for medicinal products through collaborative evidence generation. This entails fostering cooperation among stakeholders to ensure that assessments are grounded in the best available science. Addressing unmet medical needs is another crucial aspect, with a specific focus on the use of Horizon scanning to identify therapies that can meet these needs. Additionally, the strategies stress the importance of early planning, method development, and clinical evaluation support to facilitate the development of innovative therapies. Furthermore, these strategies aim to support evidence generation that is pertinent to downstream decision-makers, including regulatory authorities, healthcare providers, and patients. Additionally, global awareness of these challenges is to be raised to promote knowledge sharing and data collection, fostering a broader understanding of these innovative therapies.

Under the guidance of the “National Research Program 2021–2027” (PNR) (5) drafted by the Ministry of University and Research, Italy addresses significant issues related to innovation and growth in the healthcare sector. In Italy, the healthcare sector represents 11% of the GDP and contributes to 10% of employment, positioning it as the country’s third-largest industry. Despite Italy’s excellence in scientific production in the biomedical, pharmaceutical, and pharmacological fields, the 2020 European Innovation Scoreboard highlighted that its overall innovation capacity lags behind countries such as France, Germany, the United Kingdom, and the Netherlands. Despite the presence of numerous spin-offs in the life sciences sector, their attraction of investments for growth has been modest, and their sustainability has been disadvantaged by the absence of a robust ecosystem and an effective management approach that takes into account the high risk and uncertainty inherent to this field.

This document contains a proposal for a comprehensive reform aimed at valorizing pre-competitive discoveries and also at fostering the bidirectional exchange of knowledge among academic institutions, research entities, and local stakeholders in order to facilitate the development of shared projects, proof of concepts, and market entries, including the utilization of CRISPR-Cas9 technology. Particularly, within the context of enhancing reliable and predictive experimental models of human diseases, the importance of utilizing organoids derived from human stem cells, both from normal and tumor tissues, and developing integrated models suitable for *in vivo* transgenesis and genomic editing with CRISPR/Cas9 is emphasized. These research endeavors promise to enhance the understanding of the genetic foundations of diseases, increase the predictiveness of preclinical studies, and reduce the use of animals in experimentation, contributing to the development of innovative therapies based on unconventional approaches, including CRISPR-Cas9, which represents a key element of this innovative perspective.

Italy’s concentrated research efforts directed toward the application of CRISPR technology in rare diseases are notably centered on the examination of two genetic disorders with a higher prevalence within the country compared to other regions worldwide. These disorders are Beta-Thalassemia and Sickle Cell Disease.

Currently, Italy is actively engaged in studies aimed at assessing the safety and efficacy of autologous CRISPR-Cas9 modified CD34+ human hematopoietic stem and progenitor cells. By exploring the potential of CRISPR-Cas9 technology on CD34+ cells, the focus is on enhancing our understanding of the safety profile and effectiveness of these modified cells for therapeutic applications. This endeavor exemplifies Italy’s role in the global efforts to harness the potential of CRISPR-based approaches for addressing rare diseases and genetic disorders. By continually evaluating the progress of genetic technologies like CRISPR for rare diseases, researchers can identify potential challenges and refine their approaches to optimize patient outcomes. These efforts not only advance scientific knowledge but also pave the way for developing targeted and personalized therapies for patients affected by these debilitating rare conditions.

More importantly, a periodic review of the process will be helpful in providing the necessary perspective and framework for drug development, highlighting the opportunities for future improvements and applications in research, as well as the therapeutic and clinical considerations for their use in patients.

## 2 CRISPR therapeutic applications

The CRISPR-Cas technique offers significant advantages, enabling simultaneous modification of multiple genes with enhanced precision in DNA cutting, minimizing off-target effects (6). Recognizing target sequences through easily synthesized guide RNA molecules, CRISPR-Cas facilitates gene and genome editing in various organisms without requiring specialized knowledge or expensive equipment. Widely applied since 2013, CRISPR-Cas9 allows efficient manipulation of DNA, RNA, and epigenomes, promising revolutionary advancements in biomedical research and treatment strategies for gene-associated diseases (3).

**Figure 1:**
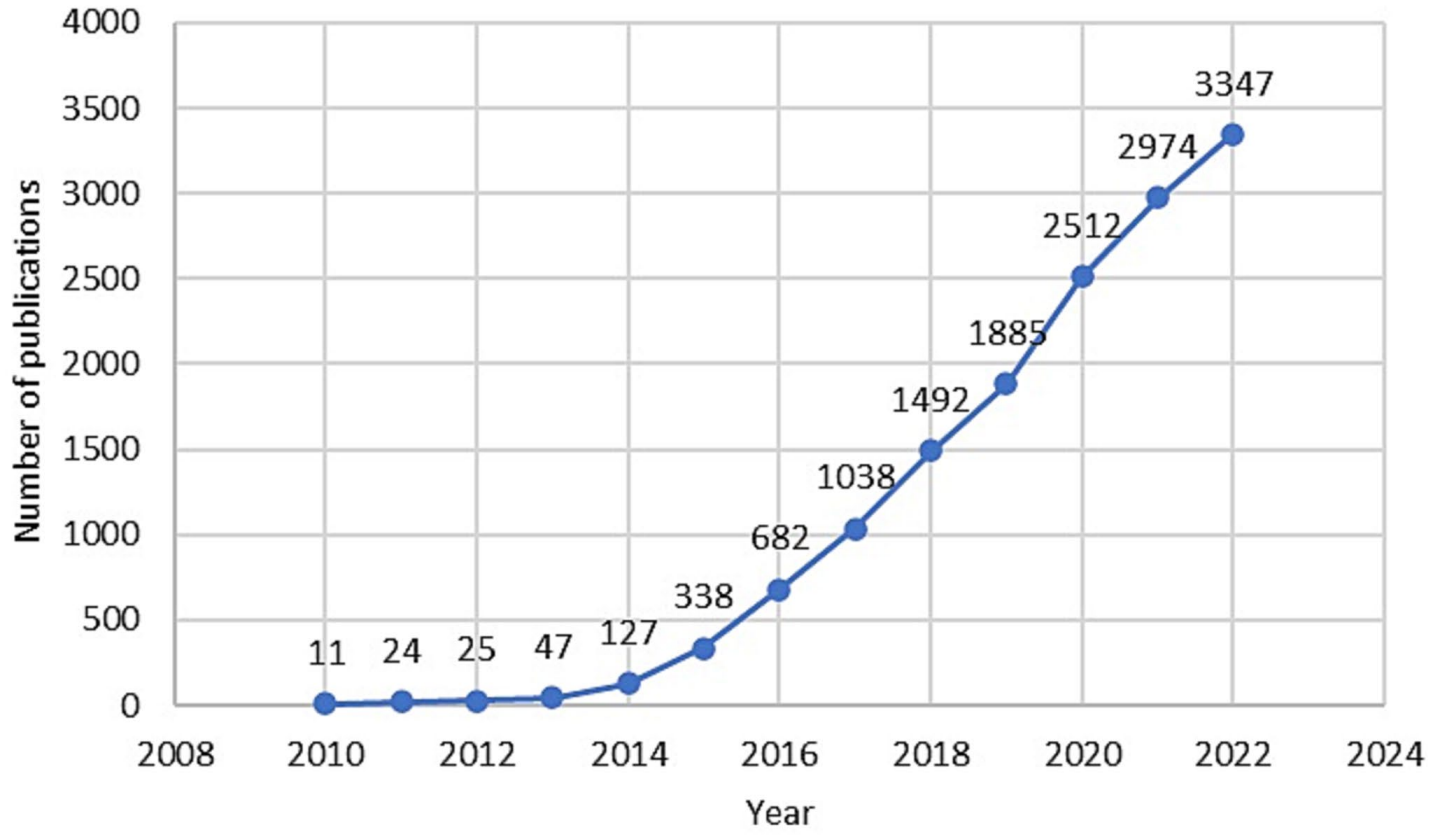
Temporal trends of publications that have CRISPR in the title and/or abstract and related to the medical area indexed in Scopus from 2010 to 2022.

Therapeutic applications derived from the CRISPR-Cas technique are many and have grown in recent years. Indeed, quick research on the Scopus search engine shows the number of publications that have CRISPR in the title and/or abstract and related to the medical area are almost 15,000. This number increased from 11 publications in 2010 to more than 3,000 only in 2022 (Figure 1).

The clinical landscape for CRISPR-Cas systems has rapidly expanded, evident in a surge of trials. As of December 2022, clinicaltrials.gov listed 71 CRISPR-related trials; 45 were human therapeutic product-focused, a significant rise from the 9 studies in 2018 (7, 8).

Based on data reported in literature (8) and/or in dedicated list, the first clinical trial based on CRISPR technology in human was initiated in 2016 at the West China Hospital, in which CRISPR/Cas9 edited immune cells were used to treat patients with lung cancer. The study aimed at evaluating the safety and feasibility of PD-1 knockout engineered T cells in treating metastatic non-small cell lung cancer with *ex-vivo* knocked-out, expanded, and selected PD-1 knockout-T cells from autologous origin (ClinicalTrials.gov Identifier: NCT02793856). After a median follow-up of 47.1 weeks, none of the 12 patients had treatment-related severe adverse events, with the conclusion that clinical application of CRISPR–Cas9 gene-edited T cells is generally safe and feasible (9).

In 2018 clinical trials of CRISPR-based genome editing therapeutics started in the US for the treatment of cancer (ClinicalTrials.gov Identifier: NCT03399448), β-thalassemia (ClinicalTrials.gov Identifier: NCT03655678) and sickle cell disease (ClinicalTrials.gov Identifier: NCT03745287) (10).

Since 2019 the drug EDIT-101 is under development for the treatment of blindness due to Leber congenital amaurosis 10 (LCA10) (11) and is designed to repair the IVS26 CEP290 mutant allele that impacts approximately 1,500 LCA10 patients in the U.S. This represents the first case of a CRISPR–Cas9 gene therapy administered directly into the patients’ bodies for a rare disease that happens to have no effective treatment currently available. The BRILLIANCE clinical trial update, currently in Phase I/II, includes safety and efficacy data from all 14 patients treated in the study to date, which includes 12 adult patients and two pediatric patients. Three out of 14 treated subjects met a responder threshold having experienced clinically meaningful improvements in best corrected visual acuity and demonstrated consistent improvements in two of the following three additional endpoints: full field sensitivity test, visual function navigation course, or the visual function quality of life.

CRISPR-Cas9 genome editing has been considered a powerful tool in cancer research. The technology can be used to investigate tumorigenesis and tumor invasion. Encouraging studies suggest that CRISPR-Cas9 is valuable for the rapid development of cancer immunotherapy (12).

Despite the increasing maturity of CRISPR-Cas9 technology, its safety and efficiency are important concerns requiring comprehensive studies. Since gene editing approaches are still in an early phase of clinical development and no CRISPR-based therapy has been approved for use in patients yet, their safety and associated clinical risks are uncertain (13). Long-term safety considerations in CRISPR/Cas9 genome editing involve challenges in detecting potential adverse effects due to factors like low editing efficiency, limited edited cell numbers, and relatively short post-editing monitoring periods. Tumorigenesis is a significant concern if off-target edits affect oncogenes or tumor suppressor genes, with tumor development potentially delayed after the initial edit. Large DNA deletions at target sites are another safety concern, ranging from hundreds to thousands of base pairs, and the exact mechanism behind these deletions is not yet fully understood. Large deletions occurring in the vicinity of Cas9 cleavage sites can affect the function of nearby genes, raising further safety concerns, especially when critical genetic elements are involved in the deleted regions (14). Understanding the molecular mechanisms behind these events is crucial for risk mitigation.

As can be read in the document dated April 28, 2020, EMA/CAT/94295/2020, EMA’s Committee for Advanced Therapies (CAT) is cautioning against the use of unregulated cell-based therapies due to safety and effectiveness concerns (15). While established medical practices use cells and blood for transplantation, if cells serve a different function in the recipient than in the donor or undergo substantial manipulation, they aren’t considered transplants and require regulation as medicinal products in the EU. Advances in genome editing techniques such as meganucleases, zinc finger nucleases, TALENs, and CRISPR/Cas9 have made genome editing more cost-effective, precise, and efficient. These innovative therapies hold promise for treating genetic diseases but entail complex development, manufacturing, evaluation, and accessibility challenges for patients.

CRISPR’s simplicity has been central to widespread adaptation of this technology but the potential dangers of CRISPR call for ethical and safety recommendations to guide development and use of this technology. The safety evaluation of CRISPR trials, or any clinical trials involving gene editing technologies, involves a rigorous and multi-step process to ensure the well-being of participants and to minimize potential risks. Overall, the safety evaluation of CRISPR trials involves a combination of preclinical research, regulatory oversight, ethical considerations, participant consent, rigorous monitoring, and thorough data analysis to ensure that the potential benefits outweigh the risks for trial participants. Further technical advances are required to ensure safe use for CRISPR application in human health such as methods to limit off-target edits (both cellular and genomic), as well as delivery systems with safety mechanisms to guard against undesirable side effects. Key safety and technical considerations include genomic and tissue specificity, efficiency of *in vivo* editing/gene modulation, along with immune system reactivity to the various components of CRISPR therapeutics (16). Genomic therapies can have a great impact on millions of people, but it is critical to ensure that human lives are not put at risk with the unsafe use of CRISPR.

## 3 History and impact of rare diseases

Research and innovations in the pharmaceutical field are most focused on the diseases and conditions with high prevalence rates.

These predominant diseases have traditionally attracted significant financial and Research and Development (R&D) resources from public and private institutions, organizations and pharmaceutical companies.

A rare disease is defined as any disease that has, in the general population, a prevalence below a given threshold, codified by the legislation of each individual country. Taken together they constitute a major health problem and involve millions of people around the world.

For many years, due to their low prevalence, rare diseases were little, if at all, attractive to the pharmaceutical industry, which understandably preferred to develop drugs aimed at diseases with large numbers of patients. It is not surprising, therefore, that fewer than 10 drugs were produced for the treatment of rare diseases from 1973 to 1983 (17) (*Orphan Drugs: Small Markets, Big Costs*, no date) (no consultable data are available regarding the type of diseases these drugs were aimed at). In 1983, in fact, US Congress passed the *Orphan Drug Act* to incentivize development of orphan drugs to meet unmet needs. The 1983 Orphan Drug Act completely changed the face of therapeutics for rare disorders. The law provided financial incentives to attract industry’s interest through a seven-year period of market exclusivity for a drug approved to treat an orphan disease, even if it were not under patent, and tax credits of up to 50% for research and development expenses. In addition, the US Food and Drug Administration (FDA) was authorized to designate drugs and biologics for orphan status (the first step to getting orphan development incentives) provide grants for clinical testing of orphan products, and offer assistance in how to frame protocols for investigations. A subsequent amendment defined a rare disease in USA as one affecting under 200,000, though a disease with more patients could qualify if the firm could not recover the costs of developing the drug.

According to the definition established by the 1999 Orphan Drug Regulation, in the European Union a disease is defined as “rare” when it affects no more than one person in 2,000 in the population considered. Rare diseases currently affect 3.5–5.9% of the world’s population. While 72% of rare diseases are genetic (for example, Adrenoleukodystrophy, Ehlers-Danlos syndrome, Osteogenesi imperfecta) (18), others are the result of bacterial (melioidosis) or viral infections (Monkeypox, Subacute sclerosing panencephalitis), allergies (Steven-Johnson syndrome) and environmental causes (mesothelioma). Besides their etiology (genetic or acquired), rare diseases are also characterized in terms of pathogenesis and evolution, and they can be degenerative (Duchenne muscolar dystrophy) and proliferative (tuberous sclerosis). In addition to this classification, the UK National Institute for Health and Clinical Excellence (NICE) also defines what are called ultra-rare diseases, which affect less than 1 patient in 50,000 people (19).

The estimated number of distinct rare diseases ranges between 7,000 and 8,000, but this number may be growing with the progress of science and research, especially with the discovery of new genetic conditions.

In Italy, the concept of “rare diseases” is defined by the Ministerial Decree of May 18, 2001, No. 279, known as the “Regulation establishing the National Network of Rare Diseases and exemption from the cost participation of related health services.” This decree identifies rare diseases, provides for the establishment of a dedicated care network to develop prevention actions, activate surveillance, improve interventions for diagnosis and therapy, promote information and training and establishes the National Register of Rare Diseases, in line with the specific provisions of the National Health Plan for the triennium 1998–2000 (20).

The law defines rare diseases as conditions that affect less than 5 people out of 10,000 in the general population. According to Orphanet, in Italy there are about 2 million rare disease patient (21): in 70% of the cases they concern pediatric patients. Based on data coordinated by the National Register of Rare Diseases of the “Istituto Superiore di Sanità” (ISS) (22), in 2020 in Italy there were an estimated 20 cases of rare diseases every 10,000 inhabitants and every year about 19,000 new cases are reported by the over 200 health facilities spread throughout the country.

Based on the 2022 MonitoRare report, there are more than 414,000 people with rare diseases (PwRDs) registered in RRDRs at the end of 2021 (+37,000 units compared to the previous year, a clear sign both of the increasingly structured process of registries coverage and tracking PwRDs in all Italian territories, and of the consolidation over time of the effect of the newborn screening policies initiated over the years) (23). The most prevalent group is diseases of the central and peripheral nervous system (16.2%), followed by the group of congenital malformations, chromosomopathies and genetic syndromes (14.3%), then diseases of the blood and hematopoietic organs (13%); all other groups of diseases record a percentage weight in the total of less than 10%.

The differences by age are very significant: in children/youth, more than 38% of rare diseases are ascribable to the group of “Congenital malformations, chromosomopathies and genetic syndromes,” the percentage weight of which decreases to less than 9% in adults for whom the modal class turns out, on the other hand, to be the group of “Diseases of the central and peripheral nervous system” (slightly less than 18%).

### 3.1 The Italian “rare diseases” scenario

The attention toward rare diseases has developed in Italy since the 90s thanks to the Piano Sanitario Nazionale (1998–2000) whose health objectives include the surveillance, prevention and support for research on rare diseases, and has grown in recent years, together with the awareness that, from a public health perspective, these diseases raise a number of problems and require specifically addressed policies.

In 2001, Italy approved Decreto Ministeriale No. 279/2001, which established the initial list of rare diseases eligible for exemption from healthcare payments. Rare diseases qualify for exemption based on specific criteria, including rarity (defined by a prevalence threshold of <5/10,000 inhabitants at the European level), clinical severity, degree of disability, and cost implications (24, 25). Two additional specific criteria have been added: diagnostic difficulties and/or delayed diagnosis, as well as challenges in finding appropriate services to address complex healthcare needs.

This policy ensures that individuals with rare diseases in Italy can access necessary healthcare without incurring significant financial burdens, and the criteria for eligibility are periodically reviewed and updated.

The Ministry of Health drew up a list of rare diseases, and those affected are exempt from contributing to health care expenditures. Currently, the ministerial list includes 453 exemption codes, which represent at least 682 specified diseases. Detection of a citizen suffering from a rare disease and therefore entitled to have an exemption is carried out by an expert clinician working at a qualified center, recognized as such by the regional health authority. The exemption is registered with a 3-letter, 3-digit alphanumeric code, which uniquely identifies the rare disease.

In order to contribute to national and regional planning of interventions aimed at the protection as well as implementing surveillance of people with rare diseases, a monitoring system has been established in Italy since 2001 through the National Rare Disease Registry (NRDR) (26) and regional and/or interregional registries (RRDRs).

Therefore, a part of the data collected through the RRDRs feeds the NRDR to obtain national epidemiological surveillance of rare diseases, perform estimates of occurrence for individual rare diseases, analysis of the performance of centers of expertise, carry out further specific studies and analyses, and, more generally, enable better governance of the National Rare Disease Network (27).

Based on the data currently included in RD registries, the number of people with exempt rare diseases in Italy is expected to reach more than 600,000 with an estimated prevalence of 1.08% in the population.

It is not easy to derive prevalence data from other regional registries, or from the national one, as variability comes from the commitment and effort of individual regions. As for the most up-to-date and available data, the federation of associations dealing with rare diseases, UNIAMO, annually produces the MonitoRare report (28), in which estimated prevalence data in Italian regions are reported (Table 1). Estimated population prevalence of people registered in RRDRs as of December 31, 2021 rises to 0.75% (0.89% in under-18s) from 0.30% in the first edition of the MonitoRare Report in 2015. At present, comprehensive and updated data for the year 2022 is not accessible or obtainable for inclusion in this study. The latest available dataset for patients registered in the RRDR is as of December 31, 2021.

**Table 1:** Estimated maximum number of people with rare diseases living in the Italian region as of December 31, 2021.

Italian region/AP	PwRD residents included in the RRDR as of 31/12/2021	Resident population as of 31/12/2021	Estimated MAX PwRD residents as of 31/12/2021	PwRD residents per 1,000 inhabitants
Abruzzo	9,298	1,273,660	11,718	7,295
Basilicata	1,288	539,999	4,968	2,385
Calabria	13,834	1,844,586	16,970	7,500
Campania	27,769	5,590,681	51,434	4,974
Emilia-Romagna	37,271	4,431,816	40,773	8,409
Friuli-Venezia Giulia	7,437	1,197,295	11,015	6,218
Lazio	42,864	5,715,190	52,580	7,516
Liguria	9,072	1,507,438	13,868	6,022
Lombardia	93,155	9,965,046	91,678	9,353
Marche	7,064	1,489,789	13,706	4,739
Molise	512	290,769	2,675	1,757
Piemonte	45,883	4,252,279	39,121	10,805
AP Bolzano	4,810	535,774	4,929	8,985
AP Trento	4,570	542,158	4,988	8,428
Puglia	24,460	3,912,166	35,992	6,248
Sardegna	10,319	1,579,181	14,528	6,523
Sicilia	17,144	4,801,468	44,174	3,567
Toscana	34,877	3,676,285	33,822	9,490
Umbria	6,447	859,572	7,908	7,502
Valle d’Aosta	267	123,337	1,135	2,168
Veneto	42,594	4,854,633	44,663	8,780
Total	440,395	58,983,122	542,645	
Prevalence	0.75%		0.92%	

AP, Autonomous Province; PwRD, person with rare disease.

Substantial disparities are evident in the prevalence of individuals with rare disease among various Italian regions and autonomous provinces. For instance, Piemonte exhibits the highest value with 10,805 residents with disabilities per 1,000 inhabitants, while Sicily registers one of the lowest values at 3,567. Certain regions, such as Lombardia and Piemonte, have significantly higher values compared to others. This variation may reflect differences in the distribution of disabilities across the country or variations in disability registration practices. Northern Italian regions (e.g., Lombardia and Emilia-Romagna) tend to have higher values compared to southern Italian regions (e.g., Sicilia and Calabria). This geographic variation could be attributed to several factors, including healthcare quality and economic well-being. This normalization can be useful for resource planning and the allocation of services for individuals with disabilities. Regions with higher values may need to address increased demand for care and support services for people with disabilities.

What is difficult to obtain is to estimate an incidence figure, either overall or by individual disease or group of diseases. In fact, the registries record when the disease was certified, which does not necessarily coincide with the date of disease onset. So, it is possible to determine for each condition when the certification was written and then to calculate year by year global and individual disease certifications or rather for each the 16 of the diagnostic categories defined by the International Classification of Diseases.

Numerous projects and initiatives, both national and international, have since developed and are being developed, sharing a common purpose: starting from scientific research to providing concrete answers to people with rare diseases and their families. The activities involve different areas from translational research to quality-of-life studies, to monitoring the national network of rare diseases. In light of the challenge that these diseases pose, both from a scientific research and surveillance perspective, in 2008 the Istituto Superiore di Sanità approved the establishment of a center dedicated entirely to rare diseases, the National Center for Rare Diseases (NCRD) (29). It did not simply inherit the previous activities, but expanded them, enhancing some aspects and making some innovations, and thus becoming one of the main scientific interlocutors in the national and international research and public health initiatives, including through coordination and participation in numerous national and international projects.

## 4 CRISPR applications in the treatment of rare diseases

There are several factors that often complicate the development of new therapeutic approaches for patients with rare diseases. For example, rarity of their occurrences hampers the advances in scientific research and delays medical referrals to disease experts who can correctly diagnose the disorder through specialized biochemical and genetic testing. Rare diseases often have limited clinical information and inadequate numbers of patients to calculate statistical significance and design clinical trials. Lack of biomarkers to evaluate the disease progression contributes to delays in treatments. Many rare diseases have early developmental or neonatal onsets resulting in poor prognosis without immediate therapeutic intervention (30).

As CRISPR-Cas9 targets specific DNA sequences, the technology works well for monogenic diseases, that is, diseases caused by mutations in a single gene. With more than 80% of rare diseases caused by genetic mutations (31), the question arises as to whether CRISPR-Cas9 could help advance the management of rare diseases.

CRISPR-Cas9 clinical applications depend on successful delivery of agents (CRISPR-Cas9 material) to the target cells. These agents are typically delivered to target cells through viral vectors, such as adeno-associated virus (32). But viral delivery systems can be immunogenic and have restrictions on the size or number of agents that can be delivered. Non-viral systems, such as liposomes, may be able to offer safer delivery systems but they have been limited by factors such as poor efficiency and reproducibility (33).

The clinical potential of treating rare disease has recently been highlighted by initial results released from a Phase 1 clinical trial (NCT04601051) treating transthyretin amyloidosis. Transthyretin amyloidosis is a rare and fatal disorder caused by misfolded forms of transthyretin protein accumulating in tissues, particularly in the nerves and heart. The design of a CRISPR/Cas9 treatment system that is delivered by lipid nanoparticles, termed “NTLA-2001,” reduced transthyretin levels by around 87%, with mild adverse effects in 3 of the 6 patients treated (34).

The results of the study also address concerns over the ability of CRISPR-Cas9 to alter non-target DNA sequences, leading to off-target effects, and introducing pathogenic variants during DNA repair. These assessments identified only “low-risk” alterations but state that long-term follow is required to monitor safety of participants.

Advances in CRISPR-Cas9 systems have helped researchers develop models and therapeutic strategies for treating rare diseases. With initial findings of a clinical trial study showing that CRISPR-Cas9 is capable of targeting proteins linked to transthyretin amyloidosis, CRISPR-Cas9 may soon offer a viable strategy for treating many types of rare disease.

Several other clinical trials on rare disease disorders using CRISPR method-derived therapies are being studied (Table 2). These conditions include Beta-Thalassemia and transfusion-dependent-thalassemia, Hemophilia B, Hereditary Angioedema, Herpes Simplex Virus Refractory Keratitis, Leber Congenital Amaurosis, Mucopolysaccharidosis, and Sickle Cell Disease.

**Table 2:** CRISPR clinical trials in Italy.

Study title	Status	Conditions	Interventions	Phase	Population	Dates
Evaluation of efficacy and safety of a single dose of CTX001 in participants with transfusion-dependent- thalassemia (TDT) and severe sickle cell disease (SCD)	Recruiting	-Beta-thalassemia -Thalassemia -Hematologic diseases -Genetic diseases, inborn -Hemoglobinopathies -Sickle cell anemia -Sickle cell disease	Biological: CTX001	Phase 3	Enrollment: 12 Age: 12–years (child, adult)	Study Start: August 2, 2022 Study Completion: February 2025
Evaluation of safety and efficacy of CTX001 in pediatric participants with severe sickle cell disease (SCD)	Recruiting	-Sickle cell disease -Hydroxyurea failure -Hydroxyurea Intolerance -Hemoglobinopathies -Hematological diseases	Biological: CTX001	Phase 3	Enrollment: 12 Age: 2–11 years (child)	Study start: May 2, 2022 Study Completion: May 2026
Evaluation of safety and efficacy of CTX001 in pediatric participants with transfusion-dependent-thalassemia (TDT)	Recruiting	-Beta-thalassemia -Thalassemia -Genetic diseases, inborn -Hematologic diseases -Hemoglobinopathies	Biological: CTX001	Phase 3	Enrollment: 12 Age: 2–11 years (child)	Study start: May 3, 2022 Study Completion: May 2026
A safety and efficacy study evaluating CTX001 in subjects with transfusion- dependent-thalassemia	Active, not recruiting	-Beta-thalassemia -Thalassemia -Genetic diseases, inborn -Hematologic diseases -Hemoglobinopathies	Biological: CTX001	Phase 2 Phase 3	Enrollment: 45 Age: 12–35 years (child, adult)	Study start: September 14, 2018 Study Completion: August 2024
A safety and efficacy study evaluating CTX001 in subjects with severe sickle cell disease (SCD)	Active, not recruiting	-Sickle cell disease -Hematological diseases -Hemoglobinopathies	Biological: CTX001	Phase 2 Phase 3	Enrollment: 45 Age: 12–35 years (child, adult)	Study start: November 27, 2018 Study Completion: October 2024
A long-term follow-up study in subjects who received CTX001	Enrolling by invitation	-Beta-thalassemia -Thalassemia -Sickle cell disease -Hematologic diseases -Hemoglobinopathies -Genetic diseases, inborn -Sickle cell anemia	Biological: CTX001	N/A	Enrollment: 114 Age: 2 years and older (child, adult, older adult)	Study start: January 20, 2021 Study Completion: September 2039

Status description. Recruiting: The study is currently recruiting participants. Enrolling by invitation: The study is selecting its participants from a population, or group of people, decided on by the researchers in advance. These studies are not open to everyone who meets the eligibility criteria but only to people in that particular population, who are specifically invited to participate. Active, not recruiting: The study is ongoing, and participants are receiving an intervention or being examined, but potential participants are not currently being recruited or enrolled.

## 5 Research strategy

CRISPR-Cas editing systems have created new avenues to study the less understood rare medical conditions. Simultaneously, it has helped accelerate the transition to clinical trials for conditions with well-characterized causal mutations. CRISPR-mediated gene editing and modulation enables scientists to create cell models to recapitulate diseases closely.

Several ongoing and upcoming clinical trials focus on CRISPR-Cas editing to either correct the mutation or functionally complement the defective protein. Early results from some of these trials have been very encouraging.

The present search for the number and type of clinical trials using CRISPR technology was conducted on the public database at clinicaltrials.gov, which is a searchable registry and results database of federally and privately supported clinical trials conducted in the United States and around the world, giving information about a trial’s purpose, participants, locations and type of intervention/treatment (35).

Specifically, the keyword “CRISPR” was used in the “Other terms” search field of the “Find a study” section. This section summarizes the details of all clinical trials with CRISPR-Cas9 technology regardless of the trial status and disease types.

No filters were applied pertaining to the location of the study, nor were there any temporal limitations. The data has been updated through September 2023.

The search result shows 89 clinical studies containing the word CRISPR within the “title,” “intervention,” “study description” or in the “sponsor/collaborators” section. Therapy-oriented clinical trials cover areas inherent in the study of antibacterials, blood diseases, cancer, metabolic disorders, and other genetic conditions.

Out of these 89 studies, only 10 are found to be completed (that is, the study has ended normally, and participants are no longer being examined or treated, meaning that the last participant’s last visit has occurred).

Of the 10 completed studies, 4 appear to be interventional (a type of clinical study in which participants are assigned to groups that receive one or more intervention/treatment (or no intervention) so that researchers can evaluate the effects of the interventions on biomedical or health-related outcomes. The assignments are determined by the study’s protocol. Participants may receive diagnostic, therapeutic, or other types of interventions).

These four studies are:

- Safety and Efficacy of CRISPR/Cas9 mRNA Instantaneous Gene Editing Therapy to Treat Refractory Viral Keratitis, targeting conditions such as Viral Keratitis, Blindness Eye, Herpes Simplex Virus Infection, Cornea (36, 37).
- PD-1 Knockout Engineered T Cells for Advanced Esophageal Cancer, targeting Esophageal Cancer (38).
- PD-1 Knockout Engineered T Cells for Metastatic Non-small Cell Lung Cancer, targeting Metastatic Non-small Cell Lung Cancer (39).
- A Study of the Safety, Engraftment, and Action of NB01 in Adults With Moderate Acne, targeting Acne Vulgaris (40).

The 89 clinical trials found through the search have different locations, most notably Canada, USA, China, Europe, and Australia.

### 5.1 Italy’s role in rare diseases research

Italy is actively advancing its gene editing research through participation in six clinical trials, notably in collaboration with the Bambino Gesù Children’s Hospital in Rome. These trials involve international partnerships, including institutions in the United States and Germany. Notable U.S. collaborators include Columbia University Medical Center in New York, Atrium Health Levine Children’s Hospital in Charlotte, North Carolina, and SCRI at the Children’s Hospital at TriStar Centennial in Nashville, Tennessee. Additionally, there is collaboration with Universitätsklinikum Düsseldorf Hospital in Duesseldorf, Germany.

One significant clinical trial at the forefront of these efforts is the evaluation of CTX001’s efficacy and safety in individuals with Transfusion-Dependent Thalassemia (TDT) and Severe Sickle Cell Disease (SCD), including pediatric patients. Identified by clinicaltrials.gov with the identifiers NCT05477563/NCT05329649, these trials mark a pioneering milestone in gene therapy for SCD and Thalassemia. It’s noteworthy that while the trials represent a groundbreaking example of CRISPR-based therapy for these conditions, the roots of CRISPR editing studies trace back to 2016, emphasizing the continuous evolution and progress in the field (41). These studies are shown below in Table 2.

Italy’s participation in research has become evident through its engagement in studies focused on the treatment of two rare diseases: Beta-Thalassemia (BT) and sickle cell disease (SCD). The rationale behind the involvement of Italian researchers in these studies is rooted in the epidemiological landscape and prevalence of these hemoglobinopathies within the country.

These genetic disorders hold a distinct prevalence in regions like the Mediterranean basin, a geographical area to which Italy belongs. The autosomal recessive nature of both Beta-Thalassemia and sickle cell disease further contributes to their prominence within this region. This prevalence, combined with the advanced medical research capabilities in Italy, has positioned the country at the forefront of endeavors to advance the understanding, diagnosis, and treatment of these conditions.

By actively participating in studies dedicated to these rare diseases, Italy underscores its commitment to addressing the unique challenges posed by these conditions and fostering breakthroughs that could potentially benefit not only its own population but also individuals across the globe affected by these disorders.

### 5.2 The impact of beta-thalassemia and sickle cell disease

Beta-Thalassemia is a condition of highly variable severity; it ranges from a form called thalassaemia minor, which is mostly asymptomatic, to the most severe form, known as thalassaemia major or Cooley’s disease, a condition that involves blood transfusion dependence [transfusion-dependent thalassaemia (TDT)].

Anemia constitutes the predominant symptom, and transfusions can range from every 2–4 weeks to once every 2–3 months, depending upon the clinical severity of the disease. Repeated blood transfusion also prevents physical abnormalities associated with the bone marrow hyperactivity, which is responsible for the characteristic skeletal changes seen with Thalassaemia (42).

Worldwide, approximately 1.5% of the population, or around 90 million people, are carriers of BT, 400,000 of whom are actually affected (43). According to Orphanet, the annual incidence at the birth of symptomatic BT is estimated at 1 in 100,000 worldwide and 1 in 10,000 people in Europe.

In the world, the Republic of Maldives has the highest incidence of thalassemia, with an 18% carrier rate of the population. The estimated frequency of BT in Cyprus is up to 16%; Thailand: 1%; Iran: 5–10%; China: 3–8%; and India: 3–4%. In Italy, about 7,000 people affected by BT. Although BT is relatively rare in the USA, there are an estimated 1.25 million carriers, making up 0.4% of the population. Thalassemia affects approximately 2,000 patients living in the USA, with 1,000 patients having BT major, but data are largely influenced by factors such as population migration. In Italy, about 7,000 people affected by BT (44). The Piera Cutino Association affirms that there are about 3 million healthy carriers in Italy and 400,000 in Sicily (45).

Currently available treatments for the management of patients with TDT and non-TDT include blood transfusion, splenectomy, hydroxyurea, iron chelation therapy, and, for a subset of patients, hematopoietic stem cell transplantation (46).

Sickle cell disease (also known as drepanocytosis) is a genetic disorder with an autosomal recessive trait. The mutation affects the β-globin chain, which is one of the component parts of hemoglobin (Hb), in which the substitution of a single amino acid results in the HbS form. Under conditions of reduced oxygen concentration, hemoglobin S precipitates inside the red blood cell, turning it into a small sickle: having become rigid, it is unable to pass through the small vessels, resulting in the cessation of circulation. This red blood cell dysfunction, in addition to aggravating anemia, activates the cascade of certain proteins, including p-selectins, adhesion molecules that cause blood cells to adhere to each other and to blood vessel walls, forming small blood clots and creating a chronic inflammatory state. This results in intense bone pain in the areas where oxygen supply is lowest, namely in the limbs (possible necrosis of the femoral head) and spine. These are so-called vaso-occlusive crises, which require multidisciplinary management as they can also occur in other organs: the brain (with cerebral ischemia or infarction), the liver (with liver failure) or the lungs (with respiratory failure). The onset of sickle cell anemia is in accordance with its severity, but usually occurs around 5–6 years of age (47).

Estimates, probably underestimated as not all patients receive a diagnosis, are about 6.5 million people affected worldwide (with 300 million carriers) (48) and 7,977 SCD patients in Italy in 2018 (of whom 1,690 under-18 and 6,287 adults) (49), where the most affected region is Sicily, where it is also common to observe an overlap with thalassemia genes, leading to thalassa-drepanocytosis.

Therapy is primarily prophylactic, aimed at containing factors that may trigger a crisis. Support with folic acid and complete vaccine prophylaxis is provided. Blood transfusions are evaluated in the case of symptomatic anemia and in cases at higher risk of complications.

Venocclusive crises can be very painful, and therefore intervention with adequate analgesic treatment and appropriate supportive therapy is necessary.

Hydroxyurea is frequently used, as it can increase the percentage of fetal hemoglobin, and consequently decrease the percentage of abnormal hemoglobin, resulting in a reduced risk of complications.

Bone marrow transplantation could be decisive: it is being carefully evaluated for young people with compromised quality and prospects of life, balancing the benefits and risks of the procedure, which remain significant.

Life expectancy is normal for subjects with beta-thalassemia minor, while it is variable for those with beta-thalassemia intermedia.

Life expectancy is reduced in subjects with beta-thalassemia major mainly due to complications from chronic transfusions (50). As for sickle cell disease, since it is a congenital disease with a chronic and aggravating course, in the absence of adequate treatment, there may be an impact on the life expectancy of patients, which, even in developed countries, is about 20 years lower than in the general population (51).

For both diseases, a crucial contribution is provided by prevention programs such as population screening, parental education, pre-natal diagnosis, and genetic counseling, which support raising awareness of the severity and complications of the disease and have been successfully implemented in several disease- prone areas worldwide (52).

### 5.3 Clinical trials’ results

Formerly known as CTX001, the now called biological intervention is called CASGEVY™ (“Vertex and CRISPR Therapeutics Announce Global exa-cel Regulatory Submissions for Sickle Cell Disease and Beta Thalassemia in 2022”) (53) (exagamglogene autotemcel) and it is an investigational, autologous, *ex vivo* CRISPR/Cas9 gene-edited therapy that is being evaluated for patients with TDT or SCD, in which a patient’s own hematopoietic stem cells are edited to produce high levels of fetal hemoglobin (HbF; hemoglobin F) in red blood cells. The elevation of HbF by CASGEVY™ has the potential to alleviate transfusion requirements for patients with TDT and reduce painful and debilitating sickle crises for patients with SCD. Earlier results from these ongoing trials were published in The New England Journal of Medicine in January of 2021 (54) and updated results were shared at the annual European Hematology Association (EHA) Congress in June 2023 (55).

In December 2023, Vertex and CRISPR Therapeutics jointly announced the approval of CASGEVY™ (exagamglogene autotemcel, exa-cel) by the U.S. FDA for the treatment of SCD and TDT (“Vertex and CRISPR Therapeutics Announce US FDA Approval of CASGEVY™ (exagamglogene autotemcel) for the Treatment of Sickle Cell Disease”) (56). CASGEVY is authorized for eligible patients aged 12 and older with SCD experiencing recurrent vaso-occlusive crises (VOCs) or Transfusion-Dependent Thalassemia (TDT) when a suitable human leukocyte antigen (HLA) matched related hematopoietic stem cell donor is unavailable. Approximately 2,000 patients in the U.K. meet the eligibility criteria for CASGEVY.

Distinguished as the first FDA-approved treatment utilizing innovative genome editing technology, CASGEVY marks a significant breakthrough in gene therapy. The European Medicines Agency (EMA) supported Casgevy through its PRIority MEdicines (PRIME) scheme, providing advanced scientific and regulatory assistance to address unmet medical needs. EMA’s recommendation relied on ongoing single-arm trials in patients aged 12 to 35, assessing Casgevy’s safety. The medication’s safety was further evaluated in a long-term follow-up study and two ongoing single-arm trials, encompassing 97 adolescent and adult patients with transfusion-dependent beta thalassemia or SCD.

To ascertain Casgevy’s efficacy and safety conclusively, the company is required to submit final results from ongoing pivotal trials, expected by August 2026. Additionally, data from the ongoing long-term follow-up study and other conducted studies will contribute to the comprehensive evaluation of Casgevy’s therapeutic profile.

The ongoing Phase 2/3 open-label trials, CLIMB-111 and CLIMB-121, are designed to assess the safety and efficacy of a single dose of CASGEVY™ in patients ages 12 to 35 years with TDT or SCD, respectively. The trials are now closed for enrollment. The study is ongoing, and participants are currently receiving the intervention or are being examined. Patients will be followed for approximately 2 years after CASGEVY™ infusion. Each patient will be asked to participate in CLIMB-131 (57), a long-term-follow-up trial (ClinicalTrials.gov Identifier: NCT04208529).

CLIMB-131 is a long-term, open-label trial to evaluate the safety and efficacy of CASGEVY™ in patients who received CASGEVY™ in CLIMB-111, CLIMB-121, CLIMB-141 or CLIMB-151. The trial is designed to follow participants for up to 15 years after CASGEVY™ infusion.

The ongoing Phase 3 open-label trials, CLIMB-141 (58) (ClinicalTrials.gov Identifier: NCT03745287) and CLIMB-151 (32) (ClinicalTrials.gov Identifier: NCT03655678), are designed to assess the safety and efficacy of a single dose of CASGEVY™ in patients ages 2–11 years with TDT or SCD, respectively. The trials are now open for enrollment and currently enrolling patients ages 5–11 years of age and will plan to extend to ages 2 to less than 5 years of age at a later date. Each trial will enroll approximately 12 patients. Patients will be followed for approximately 2 years after infusion.

Patients who enroll in these trials will have their own hematopoietic stem and progenitor cells collected from peripheral blood. The patient’s cells will be edited using the CRISPR/Cas9 technology. The edited cells will then be infused back into the patient as part of an autologous hematopoietic stem cell transplant (HSCT), a process which involves a patient being treated with myeloablative busulfan conditioning.

Patients undergoing HSCT may also encounter side effects (ranging from mild to severe) that are unrelated to the administration of CASGEVY™. Patients will initially be monitored to determine when the edited cells begin to produce mature blood cells, a process known as engraftment. After engraftment, patients will continue to be monitored to track the impact of CASGEVY™ on multiple measures of disease and for safety.

The data presented at the European Hematology Association (EHA) Congress, in August 2022, are from 75 patients (44 with TDT and 31 with SCD) with follow-up ranging from 1.2 to 37.2 months after CASGEVY™ dosing (55).

Of the 44 TDT patients, 42 were transfusion-free with follow-up ranging from 1.2 to 37.2 months after CASGEVY™ infusion. Two patients who were not yet transfusion-free had 75 and 89% reductions in transfusion volume. TDT patients had substantial mean increases in fetal hemoglobin (HbF) and corresponding increases in mean total hemoglobin (Hb) with mean total Hb levels increasing to >11 g/dL by month 3 and maintained thereafter.

All 31 patients with severe SCD characterized by recurrent vaso-occlusive crises (VOCs) (mean of 3.9 VOCs per year over the prior 2 ears) were free of VOCs after CASGEVY™ infusion through duration of follow-up, with follow-up ranging from 2 to 32.3 months. SCD patients had mean HbF (as a proportion of total Hb) of approximately 40% by the fourth month and maintained thereafter.

The safety was generally consistent with myeloablative conditioning with busulfan and autologous stem cell transplant. All patients engrafted neutrophils and platelets after CASGEVY™ infusion. Among the 44 patients with TDT, 2 patients had serious adverse events (SAEs) considered related to CASGEVY™. As previously reported, one patient had three SAEs considered related to CASGEVY™ hemophagocytic lymphohistiocytosis (HLH), acute respiratory distress syndrome and headache, and one SAE of idiopathic pneumonia syndrome that was considered related to both CASGEVY™ and busulfan. All four SAEs occurred in the context of HLH and have resolved. One patient had SAEs of delayed neutrophil engraftment and thrombocytopenia, both of which were considered related to CASGEVY™ and busulfan, and both SAEs have resolved. Among the 31 patients with SCD, there were no SAEs considered related to CASGEVY™.

## 6 Challenges and future opportunities in exploring rare diseases with CRISPR

CRISPR gene editing holds immense potential for future therapies and disease modeling in rare genetic conditions. At a molecular level, there are risks with off-target editing that need to be evaluated and mitigated before moving to the clinic (59). Similarly, CRISPR-induced homologous recombination events may also lead to undesired changes and erroneous gene editing. Some of this can be addressed with more precise editing methods like base and prime editing.

Traditionally, developing clinical trials for rare diseases has been riddled with regulatory hurdles, inadequate knowledge base, expertise, and scaling and funding difficulties. Although CRISPR-based clinical trial development shares similar challenges, success stories from some early phase trials have been encouraging. Overall, there is increasing optimism in curing rare genetic conditions radically using CRISPR-based technology.

A new method derived from Crispr-Cas9 is being tested in two new clinical trials, and it could help against 60% of genetically derived diseases, specifically, against those involving a single nucleotide.

These include SCD and heterozygous familial hypercholesterolemia, which causes high cholesterol levels induced by the malfunction of a particular gene that regulates its level in the blood.

The purpose of the two clinical trials is to test the effectiveness of the new technique called base-editing.

Base-editing (60) is a CRISPR-Cas9-based genome editing technology that allows the introduction of point mutations in the DNA without generating double strand breaks. The scope of base-editing tools has been extensively broadened, allowing higher efficiency, specificity, accessibility to previously inaccessible genetic loci and multiplexing, while maintaining a low rate of Insertions and Deletions (InDels) (61). Base-editing is a promising therapeutic strategy for genetic diseases caused by point mutations, such as many blood disorders and might be more effective than approaches based on homology-directed repair, which is moderately efficient in hematopoietic stem cells, the target cell population of many gene therapy approaches.

Base editing was shown to be effective in some preclinical tests performed on macaque monkeys with familial hypercholesterolemia (62). The involved gene is the one encoding the protein Pcsk9, which regulates blood cholesterol levels, and base editing works by converting the nitrogenous base adenine to guanine. Inactivation of Pcsk9 appeared to reduce cholesterol levels and the risk of heart disease. In macaque monkeys, the treatment proved safe and reduced blood levels of the protein by 81%, lowering blood cholesterol levels without harmful side effects.

The clinical trial VT-1001 (ClinicalTrials.gov Identifier: NCT05398029) is an open-label, phase 1b, single-ascending dose study that will evaluate the safety of VERVE-101 administered to patients with heterozygous familial hypercholesterolemia, atherosclerotic cardiovascular disease, and uncontrolled hypercholesterolemia.

Verve Therapeutics announced that the first patient with heterozygous familial hypercholesterolemia has been treated in the clinical trial that began in July 2022 and with conclusion scheduled in December 2024 (63). The company plans to enroll a total of 40 patients with the same disease during this year and to publish the first results in 2023.

Although it is less than a decade old, CRISPR-Cas9 has demonstrated unprecedented potential to revolutionize innovation in basic science. The potential benefits of such revolutionary tools are endless. However, like any powerful tool, there are also associated risks to its application.

Although access to CRISPR technology proves to be inexpensive (for the technology itself), widely available and innovative, it could lead to prohibitively expensive applications in the field of rare diseases.

Considering the large number of rare diseases, only a small portion of them are being tested for treatment by the existing CRISPR tools. Although these tools open a new approach for the treatment of rare diseases, it is necessary to improve both the efficiency and safety of the current toolbox and delivery system for expanded usage.

Regulations for clinical research involving human subjects rely on established framework to manage research risks but the risks concern lack of appropriate supervisory infrastructure, unclear supervision of research and also over-regulation that might hinder progress (64).

Considering that the vast majority of medical treatments available for rare diseases offer merely symptomatic alleviation without targeting the underlying pathological etiology, approval of more gene therapies by regulatory authorities could become game changers for patients affected by rare diseases.

## 7 Conclusion

The rise of this new therapeutic resource stands as a milestone in medical research, opening the door to highly targeted and personalized therapies for a diverse range of pathological conditions. However, the enthusiasm yielded to such innovation is inevitably accompanied by questions of deep reach.

It is crucial to emphasize the necessity for rigorous oversight and regulation of this technology. Ethics and safety must remain at the nucleus of the decision-making process, particularly in the field of genetic manipulation. Therefore, the establishment of a dedicated regulatory body is of essential importance. This regulatory authority responsible for overseeing and regulating CRISPR applications for rare disease treatments could be a specialized commission or agency dedicated to genetic therapies. This institution should be composed of a multidisciplinary panel of experts with different roles and responsibilities, including genetics experts to evaluate the efficacy and safety of CRISPR applications and to monitor advances in genetic research; ethicists to ensure that every use of CRISPR adheres to ethical principles, including informed consent and equitable treatment of patients; clinicians to assess the effectiveness of therapies and the clinical implications of CRISPR applications; legal experts to ensure that all applications comply with national and international laws and regulations; patient representatives to ensure that patient concerns and needs are adequately represented in the decision-making process.

The regulatory authority’s duties and tasks should concern the approval or rejection of research projects and clinical studies involving CRISPR, continuously monitoring the progress of research and clinical applications, the intervention in cases of disputes or misuse of the technology, the maintenance of a comprehensive record of all applications and outcomes and the collaboration with international organizations and similar institutions to ensure that best practices are shared and implemented globally.

The careful and ongoing supervision of CRISPR technology is crucial to ensure that this powerful genetic editing technique remains oriented toward therapeutic purposes and does not deviate from these fundamental objectives. While CRISPR holds immense promise in the field of medicine, allowing for the correction of genetic mutations responsible for hereditary diseases, it is essential that it is not misused or used for non-therapeutic purposes.

Furthermore, it is of fundamental importance to promote transparency and public awareness regarding CRISPR applications. Openly communicating about progress, challenges, and successes in this field can help to keep the technology aligned with therapeutic goals and prevent abuse or unethical uses, support the potential benefits of CRISPR and prevent possible deviations from a responsible therapeutic path.

The imperative for stringent vigilance should not serve as a deterrent to the progress of research and development in CRISPR-based therapies. Instead, it should serve to guarantee the responsible utilization of this extraordinary technology for the enhancement of human health. In this way, Italy can exemplify best practices in the field of genetic therapies, furnishing efficacious solutions for rare diseases while simultaneously safeguarding the principles of safety and ethics in the application of CRISPR technology.

## Author contributions

FG: Conceptualization, Data curation, Investigation, Methodology, Project administration, Supervision, Visualization, Writing – original draft, Writing – review & editing. MC: Writing – original draft, Writing – review & editing. FM: Writing – original draft, Writing – review & editing.
